# Acute Immune-Mediated Thrombocytopenia due to Oxaliplatin and Irinotecan Therapy

**DOI:** 10.1155/2019/4314797

**Published:** 2019-11-04

**Authors:** Eric L. Tam, Padma L. Draksharam, Jennifer A. Park, Gurinder S. Sidhu

**Affiliations:** Department of Medicine, Division of Hematology and Oncology, State University of New York Downstate Medical Center, Brooklyn, USA

## Abstract

We describe a case of a 63-year-old woman with advanced colon cancer and liver metastases who was treated with fluorouracil, leucovorin, and oxaliplatin (FOLFOX) and cetuximab chemotherapy. She tolerated 13 cycles of chemotherapy without any significant hematological side effects, but after the 14th cycle, she developed melena and was admitted for severe thrombocytopenia. After supportive care, the platelet counts rapidly improved to 76,000/*μ*L. Upon initiation of FOLFIRI and cetuximab chemotherapy, she again developed rectal bleeding and severe thrombocytopenia with a platelet count of 6000/*μ*L. Lab testing was positive for oxaliplatin and irinotecan drug-dependent platelet antibodies on flow cytometry assay. Drug-induced thrombocytopenia (DITP) is associated with several classes of drugs with several proposed underlying mechanisms. Prospective studies are needed to further address different mechanisms of drug-induced thrombocytopenia.

## 1. Introduction

Drug-induced thrombocytopenia (DITP) occurs frequently but can be misdiagnosed or overlooked. In patients with cancer, many chemotherapeutic agents can cause thrombocytopenia due to bone marrow suppression; other causes of thrombocytopenia can include infection, autoimmune disease, and pseudothrombocytopenia [1, 2]. Reported chemotherapeutic agents known to trigger immune drug-related thrombocytopenia are fludarabine, dactinomycin, cisplatin, oxaliplatin, and irinotecan [3, 4].

Fluorouracil, oxaliplatin, and irinotecan have been standard cytotoxic chemotherapeutic agents in advanced colorectal cancer. Oxaliplatin is a platinum derivative that has been widely used in patients with gastrointestinal malignancies including colorectal cancers. The combination of 5-fluorouracil, leucovorin, and oxaliplatin (FOLFOX) has been shown to increase survival rates and reduce the risk of disease progression in stage III colon cancer [5]. Following FOLFOX, thrombocytopenia was noted in 70% of patients, which is usually self-limited and assumed to be related to myelosuppression from oxaliplatin [6, 7]. Irinotecan is a synthetic analog of camptothecin and often given in combination with 5-fluorouracil and leucovorin (FOLFIRI) and acts through inhibition of DNA topoisomerase I. Neutropenia and thrombocytopenia result from myelosuppression of the bone marrow and are usually mild [3, 8].

In this report, we describe a patient who developed acute immune-mediated thrombocytopenia to both oxaliplatin and irinotecan with the presence of bleeding symptoms during the treatment of metastatic colon cancer. Both chemotherapy agents were found to have positive platelet drug-dependent antibodies (DDAbs).

## 2. Case Presentation

**Figure 1:**
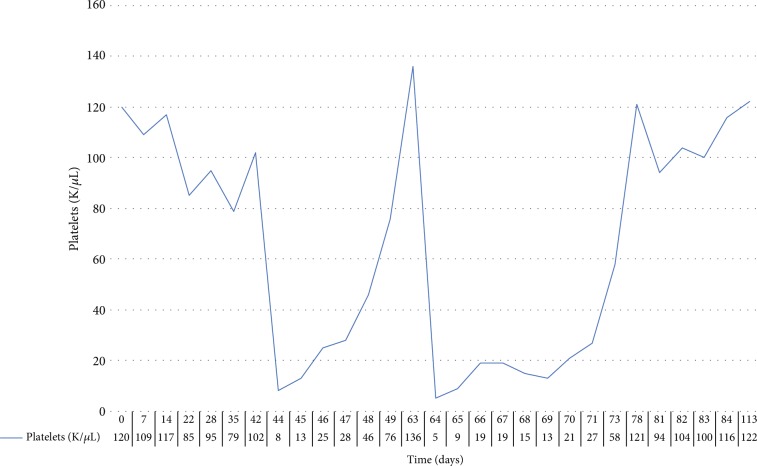
Platelet count over time for the two episodes of thrombocytopenia from oxaliplatin and irinotecan infusion with subsequent recovery.

The patient is a 63-year-old female with metastatic colon cancer (KRAS wild type) to the liver and ascites, initially treated with palliative chemotherapy with FOLFOX and Cetuximab. Her platelet count prior to initiation of therapy was 102,000/*μ*L, hemoglobin was 9.7 g/dL, and total white blood cell count was 9,000/*μ*L. An infusion of FOLFOX was administered up to the 14th cycle of treatment with minimal effects on the platelet count. The patient was tolerating treatment well with stable disease on imaging. However, two days after the 14th treatment, she developed melena. Laboratory studies obtained showed platelets of 8,000/*μ*L, hemoglobin of 8.4 g/dL, and white blood cell count of 15,790/*μ*L. She was admitted to the hospital and transfused with two units of packed red blood cells and three units of platelets with minimal response to her laboratory values. A computed tomography (CT) scan of the abdomen and pelvis showed colitis, which was treated with intravenous ciprofloxacin and metronidazole. Five days following admission, platelet levels steadily improved reaching a level of 76,000/*μ*L. One month later, she was started on second-line chemotherapy, FOLFIRI, and cetuximab. Immediately following the first cycle of irinotecan, the patient again developed rectal bleeding, and on repeat, blood work was found to have a severe drop in platelets from 136,000/*μ*L to 6,000/*μ*L within 24 hours following the completion of the FOLFIRI infusion (Figure 1).

**Figure 2:**
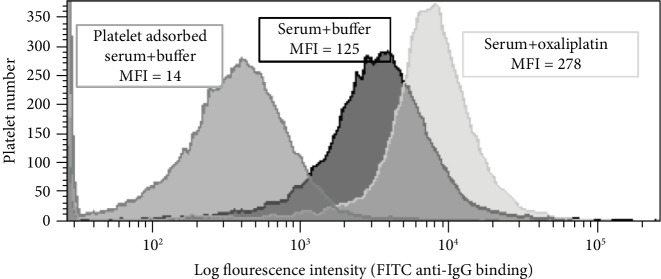
Detection of oxaliplatin-dependent platelet antibodies by flow cytometry. Platelets show high IgG binding when incubated with the patient's serum in the presence of oxaliplatin (0.1 mg/mL) (light gray histogram on the right) compared to platelets incubated with buffer/no drug present (dark histogram in the center). The numbers above each histogram are the median fluorescence intensity (MFI) values.

A peripheral blood sample was drawn immediately after the development of thrombocytopenia from FOLFIRI and sent for testing of platelet DDAbs. Drug-dependent platelet antibodies to oxaliplatin and irinotecan were detected in the patient's serum using flow cytometry techniques as previously described in the literature [6]. Interestingly, the patient's serum also showed positive reactions detected by flow cytometry in the absence of any drug but was also potentiated in the presence of oxaliplatin (Figure 2) and irinotecan. These results indicate the presence of oxaliplatin-dependent, irinotecan-dependent, and nondrug-dependent platelet-reactive antibodies. The patient's serum was also tested for fluorouracil and cetuximab-dependent platelet antibodies which were negative for both IgG and IgM DDAbs.

She was transfused with platelets and started on dexamethasone. She showed improvement, and platelet counts improved her baseline over the next few weeks. While treatment was on hold, her clinical condition deteriorated and she was not eligible for further lines of therapy. She chose to pursue the best supportive care and passed away in hospice.

## 3. Discussion

We present a case of isolated thrombocytopenia due oxaliplatin and irinotecan. Common mechanisms of thrombocytopenia in this setting include direct toxicity of the bone marrow and immune-mediated thrombocytopenia caused by drug-dependent antibodies [9]. In this case, thrombocytopenia was likely immune-mediated secondary to DDAbs. This is supported by the presentation of thrombocytopenia immediately after drug administration without evidence of any other cytopenias and laboratory conformation of oxaliplatin and irinotecan DDAbs.  Table 1 shows some of the previously reported cases in the literature of oxaliplatin and/or irinotecan-induced immune-mediated thrombocytopenia, in patients with colorectal cancer. Curtis et al. recently reported thrombocytopenia [10] induced by both oxaliplatin and irinotecan. In the first case, the patient was treated with both oxaliplatin and irinotecan for 18 cycles prior to the development of DITP. In the second case, the patient developed hemolytic anemia concurrently with DITP. Our case remains unique because DITP resulted not only from exposure to oxaliplatin with FOLFOX after 14 cycles but also upon the very first exposure to irinotecan with FOLFIRI.

In our patient, platelet DDAbs were detected in the presence of both oxaliplatin and irinotecan. This suggests that the DDAbs to irinotecan were already present prior to irinotecan exposure and the same DDAb may be implicated, or oxaliplatin generated multiple DDAbs, with one specific for irinotecan. DDAbs can induce the destruction of platelets through immune complex production, autoantibody production, or hapten-dependent antibody response [11, 12]. Metabolites of oxaliplatin are also known to be reactive and may form covalent bonds to blood proteins and macromolecules creating neoepitopes which may have specific targets such as glycoprotein IIb/IIIa platelet membrane complex, complement proteins, and ADAMTS13 [13]. Another proposed theory of historical interest hypothesizes that naturally occurring DDAbs are present on the surface of certain platelet membrane glycoproteins but with weak affinity for self-antigens without causing clinical-pathological disease [11, 12]. However, the presence of specific drugs can induce a much stronger reaction between the antigen and antibody. This may explain DITP that is noted even at low concentrations of oxaliplatin with strong antibody-platelet binding. This phenomenon may suggest that rather than a drug-induced conformational change on platelet membrane proteins resulting in neoepitopes, there is instead a high affinity binding between the drug and the complementarity-determining region (CDR) on the variable region of an antibody, which reconfigures the CDR to increase the specificity for a platelet glycoprotein binding site [14]. Our patient's serum also contained drug-independent antibodies, but we did not believe her to have idiopathic immune thrombocytopenia (ITP) since her presentation after multiple exposures (more than 10 cycles) was consistent with previously cited cases of oxaliplatin DITP.

It is of interest to further explore the mechanism behind each agent and whether the reaction represents two different mechanisms of DITP, multiple DDAbs, or if there is an antibody cross-reactivity between oxaliplatin and irinotecan which has not yet been investigated. Curtis et al. reported that complete absorption of one drug-specific antibody did not affect other DDAbs, suggesting the presence of multiple distinct DDAbs rather than a single antibody cross-reacting to different drugs. Patients treated with oxaliplatin are unusually prone to producing multiple DDAbs specific for drugs to which they are exposed. Usually, the clinical criteria are insufficient to make a diagnosis and confirmatory laboratory testing is needed. Therefore, there is a possibility that antibodies specific for additional drugs may have been present but were not checked [15]. Transfusion and pregnancy are major causes of alloimmunization, nondrug-dependent antibodies, and platelet refractoriness [16]. Pregnancy and previous transfusion can induce an HLA antibody; our patient did have a history of prior transfusion and previous pregnancies.

Better understanding of DITP will allow clinicians to readily distinguish it from other etiologies of thrombocytopenia and minimize exposure of an offending drug. Other immune-mediated diseases with oxaliplatin have been reported such as Evan's syndrome [16] and oxaliplatin-induced immune syndrome [13]. The development of multiple antibodies and antibody targets with the use of oxaliplatin carries clinical significance as it may limit the use of an otherwise versatile and effective therapeutic agent in colorectal cancer.

## Figures and Tables

**Table 1:** Known cases of oxaliplatin and irinotecan drug-induced immune-mediated thrombocytopenia. IVIG = intravenous immunoglobulin.

Study	Drug	Cycles prior to DITP	Platelet count (per *μ*L)		Time to platelet recovery (days)	Treatment
			Initial	Nadir		
Akdeniz et al. [17]	Oxaliplatin	12	151,000	20,000	6	Corticosteroids, platelets, IVIG
Bautista et al. [6]	Oxaliplatin	3	226,000	4,000	12	Antihistamine, corticosteroids, platelets
	Oxaliplatin	3	87,000	66,000	14	Corticosteroids
Buti et al. [18]	Oxaliplatin	11	117,000	17,000	NA	NA
Cobo et al. [19]	Oxaliplatin	12	202,000	7,000	NA	NA
Curtis et al. [8]	Oxaliplatin	17	150,000	6,000	7	Platelets
	Oxaliplatin	10	136,000	6,000	21	Observation
Curtis et al. [10]	Oxaliplatin, irinotecan	20	130,000	0	NA	Corticosteroids
	Oxaliplatin, irinotecan, leucovorin, dexamethasone, diphenhydramine, palonosetron	18	157,000	5,000	NA	Platelets
Earle et al. [20]	Oxaliplatin	1	99,000	6,000	14	Platelets, corticosteroids
Fontão-Wendel et al. [9]	Oxaliplatin	7	164,000	5,000	3	Corticosteroids, IVIG
James et al. [14]	Oxaliplatin	28	145,000	0	5	Corticosteroids, platelets
Koutras et al. [21]	Oxaliplatin	14	NA	NA	2	Corticosteroids, antihistamines, platelets
Mirtsching et al. [11]	Irinotecan	NA	347,000	84-100,000	NA	Observation
Mittal et al. [12]	Oxaliplatin	8	175,000	4,000	9	Platelets, corticosteroids
Ohta et al. [22]	Oxaliplatin	12	136,000	35,000	7	Corticosteroids
Pavic et al. [23]	Oxaliplatin	1	253,000	5,000	NA	Corticosteroids
Saif et al. [24]	Oxaliplatin	11	89,000	17,000	2	Platelets
Santodirocco et al. [25]	Oxaliplatin	15	124,000	21,000	5	Corticosteroids, platelets
Schade et al. [26]	Oxaliplatin	14	89,000	5,000	45	Platelets
Shao et al. [27]	Oxaliplatin	24	163,000	4,000	NA	Platelets
Suh et al. [15]	Oxaliplatin	11	113,000	3,000	60	Platelets, IVIG, corticosteroids
Suzuki et al. [28]	Oxaliplatin	23	253,000	3,000	5	Platelets, corticosteroids
Taleghani et al. [29]	Oxaliplatin	15	221,000	5,000	5	Platelets, corticosteroids
Tam et al. [this report]	Oxaliplatin, irinotecan	14	102,000	8,000	5	Corticosteroids, platelets
Curtis et al. [30]	Leucovorin, palonosetron	19	144	17	4	Dexamethasone
Pan et al. [31]	Oxaliplatin	28	NA	<5,000	4	IVIG, dexamethasone
